# Electrocorticography of Spatial Shifting and Attentional Selection in Human Superior Parietal Cortex

**DOI:** 10.3389/fnhum.2017.00240

**Published:** 2017-05-11

**Authors:** Maarten Schrooten, Eshwar G. Ghumare, Laura Seynaeve, Tom Theys, Patrick Dupont, Wim Van Paesschen, Rik Vandenberghe

**Affiliations:** ^1^Laboratory for Cognitive Neurology, Department of Neurosciences, KU LeuvenLeuven, Belgium; ^2^Neurology Department, University Hospitals LeuvenLeuven, Belgium; ^3^Laboratory for Epilepsy Research, KU LeuvenLeuven, Belgium; ^4^Neurosurgery Department, University Hospitals LeuvenLeuven, Belgium; ^5^Laboratory for Neuro- and Psychophysiology, KU LeuvenLeuven, Belgium

**Keywords:** selective attention, intraparietal sulcus, superior parietal lobule, electrocorticography, spatial shifting, invalidity effect

## Abstract

Spatial-attentional reorienting and selection between competing stimuli are two distinct attentional processes of clinical and fundamental relevance. In the past, reorienting has been mainly associated with inferior parietal cortex. In a patient with a subdural grid covering the upper and lower bank of the left anterior and middle intraparietal sulcus (IPS) and the superior parietal lobule (SPL), we examined the involvement of superior parietal cortex using a hybrid spatial cueing paradigm identical to that previously applied in stroke and in healthy controls. In SPL, as early as 164 ms following target onset, an invalidly compared to a validly cued target elicited a positive event-related potential (ERP) and an increase in intertrial coherence (ITC) in the theta band, regardless of the direction of attention. From around 400–650 ms, functional connectivity [weighted phase lag index (wPLI) analysis] between SPL and IPS briefly inverted such that SPL activity was driving IPS activity. In contrast, the presence of a competing distracter elicited a robust change mainly in IPS from 300 to 600 ms. Within superior parietal cortex reorienting of attention is associated with a distinct and early electrophysiological response in SPL while attentional selection is indexed by a relatively late electrophysiological response in the IPS. The long latency suggests a role of IPS in working memory or cognitive control rather than early selection.

## Introduction

The distribution of spatial attention is characterized by periods of spatially sustained attention alternating with transient spatial shifts. For several decades, based on patient lesion studies, models of spatial attention in the human brain have associated spatial shifting with the inferior parietal lobule, the right temporoparietal junction (TPJ) in particular (Friedrich et al., 1998; Corbetta and Shulman, 2002; for review see Vandenberghe et al., 2012). A role of TPJ has been confirmed by functional imaging studies in the intact human brain (e.g., Corbetta et al., 2000; Geng and Vossel, 2013; Gillebert et al., 2013). Contrary to what one would have predicted from lesion studies, recent functional imaging evidence in humans and nonhuman primates revealed that the medial and lateral wall of the superior parietal lobule (SPL) are robustly and consistently activated during spatial shifts (Vandenberghe et al., 2001; Yantis et al., 2002; Molenberghs et al., 2007; Caspari et al., 2015). Both in humans (Vandenberghe et al., 2001; Yantis et al., 2002) and in the nonhuman primate brain (Caspari et al., 2015), the contribution of SPL to spatial shifts is independent of the direction of the shift, leftward or rightward. Furthermore, response amplitudes do not differ between left and right SPL. The role of SPL in spatial shifting in the healthy brain does not directly relate to the severely lateralized spatial-attentional problems seen in clinical neglect. Clinical neglect commonly occurs following an ischemic lesion in the middle cerebral artery territory and SPL lies outside this territory. In nonhuman primates, the lack of an effect of the direction of attention in SPL stands in clear contrast with the attentional effects in the intraparietal sulcus (IPS) which are strongly sensitive to the direction of attention (Caspari et al., 2015), in line with the topographical organization described in IPS (Silver et al., 2005).

While a classical neglect syndrome is more severe and longer-lasting following right- compared to left-hemispheric lesions, a contralesional spatial shifting deficit can occur both with left- and with right-sided parietal lesions (Posner et al., 1984; Gillebert et al., 2011). Recent patient lesion studies of spatial shifting and contingent reorienting have confirmed the contribution of superior parietal cortex to spatial attention deficits, both IPS (Molenberghs et al., 2008; Ptak and Schnider, 2010; Gillebert et al., 2011) and SPL (Vandenberghe et al., 2012).

**Figure 1:**
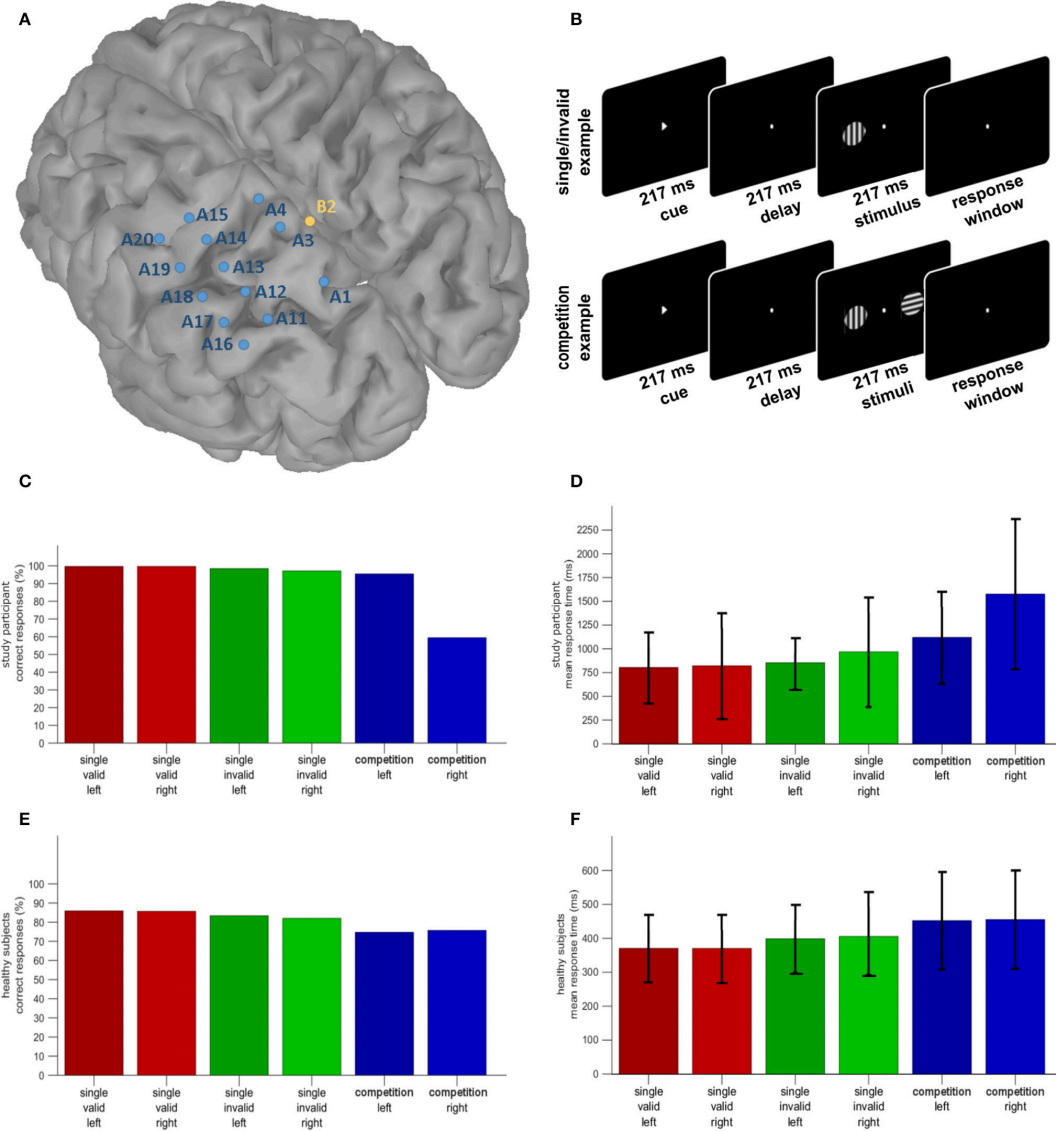
**(A)** Distribution of the electrode positions on a surface rendering of the patient's MRI. SPL and the postcentral sulcus are artificially dilated in order to better show the position of the electrodes with respect to these sulci. **(B)** Hybrid spatial cueing paradigm (Gillebert et al., 2011). **(C)** Accuracy of the study participant in the different experimental conditions. **(D)** Reaction times of the study participant in the different experimental conditions (mean and S.D.). **(E)** Accuracy in the same paradigm in a group of 22 healthy controls. **(F)** Reaction times in the same paradigm in a group of healthy controls (mean and S.D.). Note that the Y axis differs between the patient and the controls given the overall slower reaction times in the patient.

Electrocorticographic (ECoG) recordings offer an opportunity to investigate human brain function with unparallelled spatial, temporal, and spectral resolution. We report the results of a recording of the upper and lower bank of the left anterior and middle IPS and the lateral and superiomedial side of the SPL during a hybrid spatial cueing paradigm in a patient under presurgical evaluation for refractory partial epilepsy (Figure 1A). The hybrid spatial cueing paradigm was identical to that used by Gillebert et al. (2011), in patients with parietal lesions (Gillebert et al., 2011; Vandenberghe et al., 2012) and in healthy controls (Gillebert et al., 2013; Vandenberghe and Gillebert, 2013) to study spatial reorienting and attentional selection between competing stimuli (Figure 1B). Originally based on the Posner spatial cueing paradigm (Posner et al., 1980), it probes attentional selection between competing stimuli as well as attentional reorienting following invalid cues within a same experiment.

The ECoG signal was analyzed in different, complementary ways: Event-related potentials (ERP), event-related spectral perturbation analysis (ERSP), intertrial coherence (ITC), and weighted phase lag index (wPLI). The event related measures (ERP, ERSP, and ITC) offer complementary advantages to understand the neurophysiological mechanisms of cognitive tasks (Makeig et al., 2004). The ERP indicates overall stimulus-related amplitude changes with a high temporal precision that are simple and fast to compute. However, ERP may not often pick up small variations in a particular frequency band which can be of neurophysiological importance and occur at particular temporal intervals. In contrast to ERP, ERSP and ITC are based on time-frequency decomposition sensitive to power change and phase synchronizations, respectively, in a particular frequency band. ITC measures an evoked effect that results in a strong phase synchrony across trials. ITC is closely related to the ERP as the ERP depends on the ITC and the response amplitude. ERSP measures the mean change in spectral power compared to the baseline. Such a power change may or may not be picked up by ITC analysis. ERSP and ITC are not necessarily coupled and can be interpreted independently of each other. Phase-based measures like ITC are less sensitive to noise due to the random phase distribution of noise across trials in comparison to power amplitude based measures such as ERSP. Our fourth measure, the wPLI, is a measure of phase leads or lags between sensors (Stam et al., 2007). As a measure of synchronization between sensors, it is relatively invariant against the presence of common sources (e.g., volume conduction or active reference electrodes) (Stam et al., 2007).

## Materials and methods

### Subject

A right-handed 31 year old female patient with magnetic resonance imaging (MRI)-negative refractory partial epilepsy was hospitalized for a presurgical workup, including continuous video and ECoG recordings from a surgically implanted subdural grid covering the left parietal cortex (Figure 1A). She suffered from cryptogenic partial epileptic and secondarily generalized seizures starting with sensory symptoms in the right leg. EEGs, ^18^F-fluorodeoxyglucose positron emission tomography (PET) and ictal perfusion single-photon emission computed tomography (CT) all suggested a left parietal focus. Her vision was normal, as was her interictal neurological examination. Conventional neuropsychological assessment revealed normal digit span forward and backwards, normal scores on the Auditory Verbal Learning test (total learning 49/75, % delayed recall 92%), mild anomia (Boston Naming Test 42 out of 60), and scores within the normal range in the executive domain. Total intelligence quotient on the Wechsler Adult Intelligence Scale was 94. During experimental testing the patient was treated with lacosamide, levetiracetam and oxcarbazepine for her seizures, alizapride, ondansetron and methylprednisolon for postoperative nausea and paracetamol and ketorolac for headache.

The study participant provided written informed consent in accordance with the declaration of Helsinki. The experiment was approved by the Ethics Committee of the University Hospitals Leuven.

### Experimental paradigm

Stimuli were presented using Presentation 14.2 (Neurobehavioral Systems, Albany, CA, USA). The eye-screen distance was 70 cm. Testing was performed in a dimly lighted room.

The hybrid spatial cueing paradigm was identical to that used by Gillebert et al. (2011; Vandenberghe and Gillebert, 2013) in patients with parietal lesions and in healthy controls to study spatial reorienting and attentional selection between competing stimuli (Figure 1B). The patient was instructed to fixate the central fixation point and to select a left or right button depending on the orientation of the target grating, horizontal or vertical (covert orienting). In two thirds of the trials, the target grating appeared at the cued location on its own (validly cued trial) (Figure 1B). In one sixth of the trials it appeared at the cued location together with a competing distracter in the opposite hemifield (competition trial). In these trials the cue was always valid. In another sixth of the trials the target appeared at the uncued location, without distracter (invalidly cued trial). Throughout the experiment a central white fixation dot (diameter 0.27°) was present, except during the cue phase. A trial started with a central arrow cue pointing to the left or the right (217 ms duration; size 0.59° × 0.66°), followed by a delay (217 ms duration) and the test phase during which one or two sinusoidal gratings appeared on the horizontal meridian at 7.6° eccentricity (duration 217 ms; diameter 3.5°; 1.14 cycles/°). In the validly cued single-grating trials (2/3 of trials) the target stimulus appeared on its own in the cued location. In the invalidly cued single-grating trials (1/6 of trials) a single grating appeared at a location contralateral to the cued location necessitating an attentional shift. In the competition trials (1/6 of trials) two stimuli appeared in the test phase, one to each side of the fixation point. The cue and delay phase was the same between trial types. In trials with only a single grating, the subject had to respond to the single grating and the cue had a predictive value for the location of the target. In the competition trials short-term memory of the cue was necessary to determine which of the gratings was the target. Note that this differs from the clinical visual extinction test where subjects have to detect both targets under simultaneous stimulation conditions and there is no prior spatial cue. In case of two stimuli, the distracter and the target orientation differed in half of the trials.

The onset of the subsequent trial was paced by the subject's response, with a 1,650 ms interval between the patient's response and the next cue onset (Figure 1B; Gillebert et al., 2011). The patient completed 20 runs of 48 trials (960 trials in total). Conditions were balanced per run.

Eye movements were monitored using a horizontal electrooculogram (EOG). In case of any deviation the experimenter informed the subject online to maintain fixation.

Forty-four (5.1%) trials were excluded from the analysis based on the presence of saccades which occurred almost all near grating offset. There were no conditions that contained significantly more saccades although there were more saccades during invalidly cued trials with a right-sided target (11.1%) than during invalidly cued trials with a left-sided target (1.4%).

Prior to the experimental runs, the patient performed two practice runs of 48 trials with auditory feedback.

### ECoG and EOG acquisition and preprocessing

ECoG and EOG acquisition were performed with a Brainbox ECoG Amplifier EEG-1166 (Braintronics, Almere, The Netherlands) at a sampling frequency of 4,096 Hz, a resolution of 16 bit, a stopband frequency of 2,048 Hz and a stopband ripple of -40 dB, using BrainRT Software Suite version 3 patch pack 1 build 3,874 (OSG, Rumst, Belgium). Two PMT Cortac grids (PMT Corporation, Chanhassen, USA) with 3 mm platinum contacts with an interelectrode spacing of 10 mm were implanted, grid A consisting of 4 × 5 contacts points and grid B consisting of 4 × 1 contacts interhemispherically (Figure 1A, Table 1). At the time of testing, channels A2, A5, A6 to A10, B1, B3, and B4 were no longer usable due to the poor signal quality and were excluded from further analysis. The two most anterior remaining contact points (A15, A20) were located above the primary motor cortex. Electrocortical stimulation elicited a motor response. These electrodes will not be considered in the further analysis. Three adjacent contact points (A4, A14, A19) overlayed the posterior bank of the postcentral sulcus. Somatosensory stimulation elicited a response at these sites and they will also be excluded from further analysis. No motor or somatosensory responses were present in any of the remaining electrodes, which overlaid the upper and lower bank of the anterior (A13, A18) and middle segment (A11, A12, A16, A17) of the left IPS, the lateral SPL (A3, A1) and left medial parietal cortex (B2) (Figure 1A; Table 1). The last two runs had to be excluded due to poor signal quality, leaving 864 trials. A 10 mm Ag/AgCl cup electrode at position Fpz was used as the hardware recording reference. Signal processing was done on a Dell Optiplex 990 workstation running Windows 7 64-bit Service Pack 1 (Microsoft, Redmond, USA) in MATLAB 7.8.0.347 (R2009a) (The MathWorks Inc., Natick, MA, USA). ECoG and EOG data were imported into MATLAB with BRTToMatlab 4.0 (OSG, Rumst, Belgium) and downsampled to 1,024 Hz and the remaining ECoG channels were rereferenced to the average of all grid electrodes included in the analysis using EEGlab 9.0.8.6b (Schwarz Center for Computational Neuroscience, San Diego, USA). The electroencephalographic (EEG) signal was notch filtered using a Parks-McClellan notch filter and bandpass filtered between 0.15 and 500 Hz using a butterworth filter with filter order 2 and removing DC offset, as implemented in ERPlab 4.0.3.1 (UC-Davis Center for Mind and Brain, Davis, USA). Subsequently the data were epoched relative to grating onset. Baseline subtraction was performed −200 to 0 ms relative to cue onset. Epochs were included regardless of response accuracy.

**Table 1:** **MNI coordinates of the electrode positions**.

**Anatomical region**	**Electrode label**	**MNI coordinates**
Primary motor cortex	A15, A20	(−31, −27, 64), (−42, −24, 63)
Postcentral sulcus	A4, A14, A19	(−12, −40, 67), (−33, −36, 65), (−43, −35, 58)
Medial SPL	B2	(−1, −57, 59)
Lateral SPL	A3, A1	(−14, −51, 62), (−16, −72, 52)
Anterior IPS segment	A13, A18	(−35, −47, 61), (−45, −46, 54)
Middle IPS segment	A17, A12, A16, A11	(−46, −56, 50), (−36, −57, 56), (−46, −66, 45), (−37, −67, 52)

*For the different electrode positions, the MNI x, y, and z coordinates are provided*.

EOG data were scored for the presence of saccades using an heuristic threshold of 22 μV within a 100–600 ms time window postgrating onset.

### Imaging

Structural brain MRI was obtained on a Siemens Magnetom Aera 1.5 T MRI scanner (Siemens AG, Munich, Germany) and a Toshiba Aquilion One ViSION CT scanner (Toshiba Medical Systems Corporation, Tochigi-ken, Japan). The postoperative head CT scan was coregistered to a preoperative MRI scan by performing a rigid transformation based on the maximization of the mutual information criterion (Maes et al., 1997) using SPM8 (Wellcome Trust Centre for Neuroimaging, UCL, London, UK). Electrode positions were determined on the coregistered postoperative CT. The coregistered CT-MRI and the electrode positions were normalized to Montreal Neurological Institute (MNI) space using SPM8. The MRI was segmented using BrainSuite 14b (build #1975) (Shattuck and Leahy, 2002). Electrode positions are visualized in Figure 1A using Brainstorm (Tadel et al., 2011; http://neuroimage.usc.edu/brainstorm) and the MNI coordinates corresponding to the electrode positions are provided in Table 1.

### Behavioral analyses

Performance in the patient was compared to that of a group of 22 healthy controls from a previous study performing a highly similar paradigm. The controls had to discriminate the orientation of the target grating. In the controls, the target grating could have an orientation of 45° minus or plus × °. The value of x was titrated so as to reach an accuracy of about 80–85%. In order to test for condition-dependent differences between the patient and controls, the revised standardized difference test was used (Crawford and Garthwaite, 2005). A Crawford-Howell modified *t*-test was used to compare individual conditions between the patient and the control group (Crawford and Garthwaite, 2005).

### ECoG analysis

The main contrasts of prior interest were the contrast between invalidly and validly cued target trials (invalidity effect) and the contrast between competition trials and validly cued single-grating trials. The interaction effect between validity and direction of attention was also determined. Further effects of direction of attention were also determined: The effect of the direction of the cue, leftward or rightward from cue onset till grating onset, as well as the effect of the direction of attention from grating onset in the competition trials.

For the different contrasts the average evoked potentials were compared. Huynh-Feldt and Greenhouse-Geisser were used to test for sphericity. Direct comparisons between two conditions were carried out by means of a two-sided Student's *t*-test assuming equal variances repeated for every datapoint. Factorial analyses were carried out by means of two-way ANOVA for unbalanced design. The statistical significance threshold was set at *P* < 0.05 after Bonferroni correction for the number of electrodes (*n* = 9), with the additional requirement that significance had to persist for a continuous time period of at least 10 ms. Adjacent time points are highly correlated and distant time points are not. As such Bonferroni correction in the time domain is not suited (not a form of repeated independent testing) and a time criterion is preferable. In the space domain the effects on the individual electrodes (space) are less dependent, but not totally independent. Bonferroni correction is used in order to select the most robust effects, although it could be argued that this method of correction is too stringent.

ERSP analysis allows to determine the event-related power in the spectrotemporal domain (1–150 Hz). ERSP was calculated by means of the EEGlab newtimef() function in the frequency range 1–150 Hz at every 2 Hz using fast Fourier transforms and Hanning window tapering. When ERSP revealed differential effects between conditions, each condition was compared to baseline in order to determine whether the difference was due to either increased synchrony in one condition or increased desynchronization in the other condition compared to baseline. Hence, the terms (de)synchronization in the results section are based on the contrast between each of the experimental conditions in combination with the contrast of the experimental condition with baseline.

For the sake of comparison with previous ECoG studies of the Posner spatial cueing paradigm (Daitch et al., 2013) we also performed an ITC analysis within the theta frequency range. ITC, a phase locking factor, indicates a strength of phase alignment across trials at each time and frequency bin with magnitude scale 0 (weakest) to 1 (strongest). ITC was estimated along with ERSP using EEGlab newtimef() function with the same parameter settings as for ERSP.

The significance levels of the ITC and ERSP were tested by bootstrap re-sampling method. The spectral estimates of a single trial from different time windows of the baseline period were sampled 1,000 times. This produced a baseline distribution and its percentile values were used as the threshold mentioned. Statistical significance of a contrast of conditions was evaluated based on 1,000 random permutations of the trials across conditions keeping the total number of trials in the dataset unchanged. Significance of the condition and contrast were set at *P* < 0.05 corrected for the number of electrodes (*n* = 9).

To study connectivity between time series from the different channels, the wPLI was calculated (Vinck et al., 2011). The wPLI analysis was performed for all 36 possible connections between the nine electrodes. The direction of the connection was interpreted based on the sign of wPLI value. The Phase Lag Index is a measure of phase leads or lags between sensors (Stam et al., 2007). The weighting factor in wPLI is the magnitude of the imaginary component of the cross-spectrum (Vinck et al., 2011). wPLI is less sensitive to noise sources and has increased statistical power compared to PLI (Vinck et al., 2011). wPLI was calculated as follows: The spectral power of the ECoG signals was estimated using the periodogram based Welch algorithm with a moving Hanning window of 500 ms with 50% overlap. Based on spectral power peaks and local maxima identified across all frequency bins and channels, two frequency bands were selected: 6–10 Hz and 15–20 Hz. The data in these frequency bands were narrow bandpass filtered. After a Hilbert transform, cross-spectral density (CSD) between two complex signals y_*i, n, k*_ and y_*j, n, k*_ of channels *i* and *j* for each frequency band was calculated for each time point *n* and each trial *k* as:

(1) $$\begin{array}{r} {\text{CSD}}_{i,j,n,k}\;={\text{y}}_{i,n,k}{\text{y}}_{j,n,k}^{*} \end{array}$$

where ^*^ is the complex conjugate.

wPLI was calculated across trials at each time point *n* according to:

(2) $$\begin{array}{r} {\text{wPLI}}_{\left(i,j,n\right)}=\frac{\left(1/K\right)\overset{K}{\underset{k=1}{\sum }}ℑ\left(\text{CS}{\text{D}}_{i,j,n,k}\right)}{\left(1/K\right)\overset{K}{\underset{k=1}{\sum }}|ℑ\left(\text{CS}{\text{D}}_{i,j,n,k}\right)|} \end{array}$$

where ℑ indicates the imaginary part of CSD and K equals the number of trials.

wPLI was calculated for each condition separately. Statistical significance of a pairwise contrast of conditions was evaluated based on 2,000 random permutations of the trials across datasets keeping the total number of trials in the dataset unchanged. Significance of the contrast was set at *P* < 0.05 corrected for the number of connections tested (*n* = 36).

## Results

### Behavioral analysis

The increase in reaction times in invalidly compared to validly cued single-grating trials was significantly larger in the patient (99 ms) than in the controls (31 ms) (modified *t* = 3.71, *P* < 0.002) (Figures 1D,F). For the invalidly cued single-grating trials, the patient was significantly impaired for right-sided vs. left-sided targets compared to controls (modified *t* = 2.33, *P* < 0.03) (Figures 1D,F). Compared to controls the patient was significantly less accurate (modified *t* = 3.27, *P* < 0.004) and slower (modified *t* = 8.69, *P* < 0.000001) for right-sided vs. left-sided targets in the competition trials (Figures 1C–F). Compared to valid single-grating trials, competition trials were responded to less accurately (*P* < 0.0001) and more slowly (*P* < 0.0001) by the healthy controls and this did not differ in the patient compared to controls (*P* > 0.1). Note that the overall difference in accuracy between the individual and the controls is not meaningful as the orientation difference in the patient was fixed at 90° while in controls the difference was titrated to obtain an accuracy around 85%.

### Effects of the direction of attentional cue

**Figure 2:**
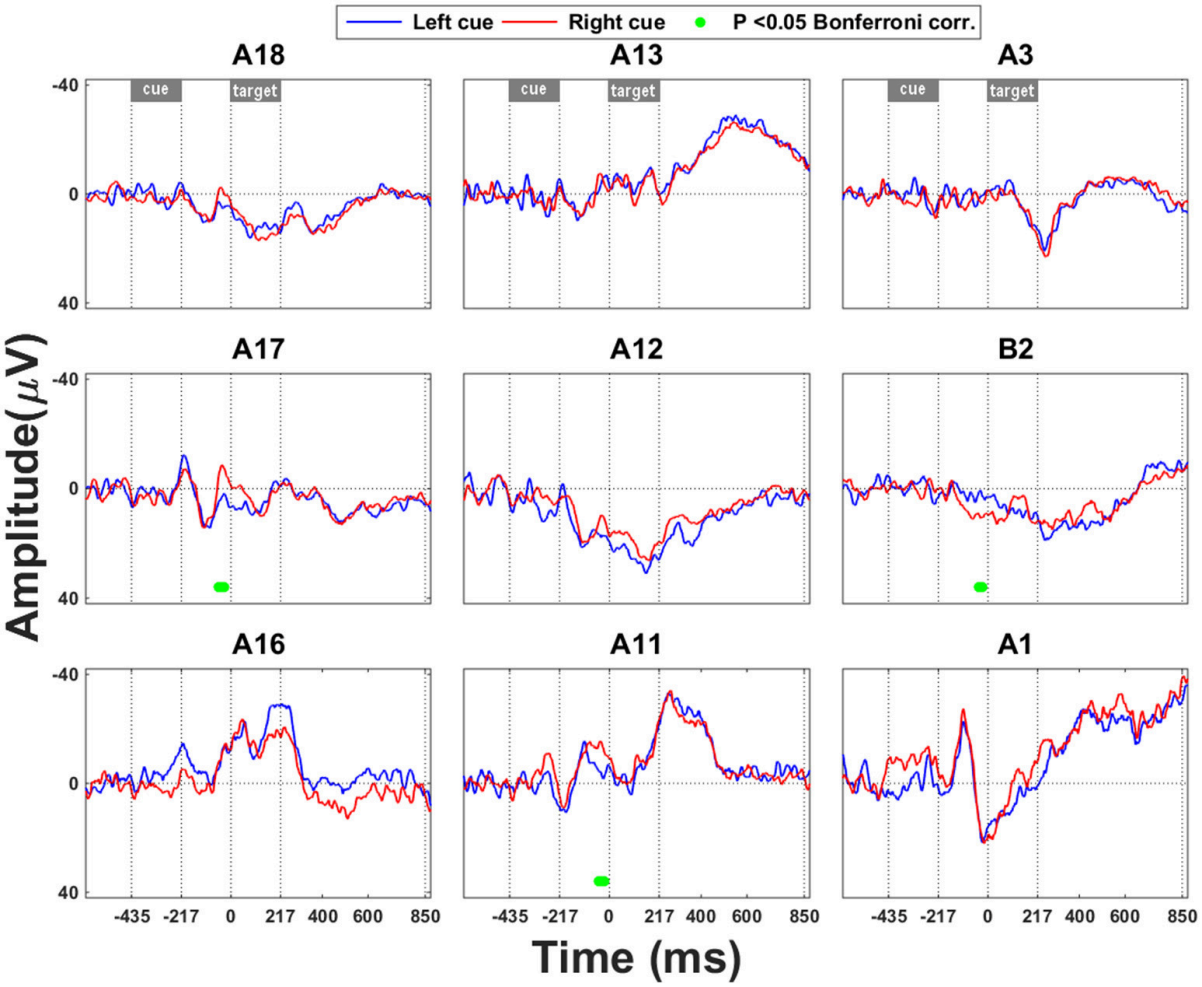
**Leftward vs. rightward cueing trials: ERP analysis**. Significant effects that occur in the interval between cue onset and grating onset are marked by a green bar. Time point 0 refers to the onset of the grating. The significance threshold is set at *P* < 0.05 corrected for the number of electrodes during a minimum continuous period of 10 ms. The plots for the different electrodes are positioned in accordance with their position on the cortical surface (Figure 1A).

The earliest effect of cue direction was seen in the most posterior IPS electrodes (A11, A17) approximately 384–390 ms after cue onset, with a negative deflection for rightward vs. leftward attention in posterior IPS (Figure 2). There was also a positive ERP in SPL for rightward vs. leftward attention with similar timing characteristics (Figure 2).

### Invalidity effect

**Figure 3:**
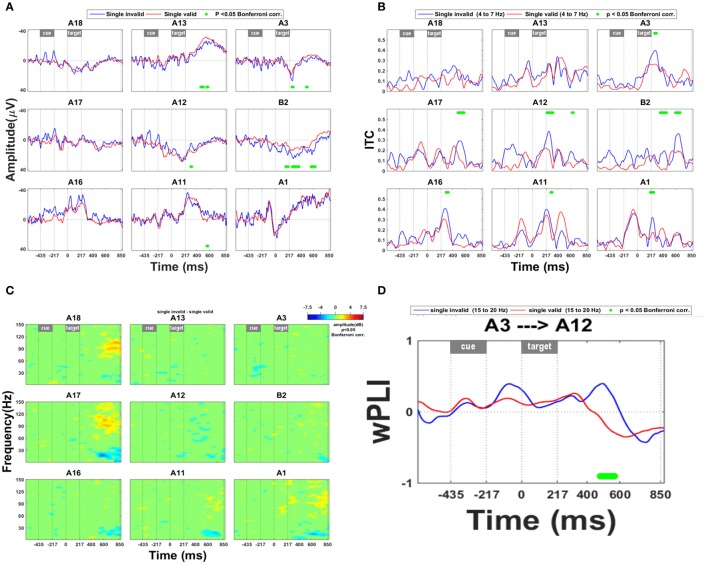
**Invalidity effect. (A)** ERP during validly cued trials and during invalidly cued trials. Significant deficits following target onset between validly cued and invalidly cued trials are marked by a green bar. The significance threshold is set at *P* < 0.05 corrected for the number of electrodes during a minimum continuous period of 10 ms. The plots for the different electrodes are positioned in accordance with their position on the cortical surface. **(B)** ITC analysis within the theta band (4–7 Hz) during invalid vs. valid cueing trials. The significance threshold is set at *P* < 0.05 corrected for the number of electrodes (*n* = 9) using a nonparametric bootstrapping approach with 1000 randomizations. **(C)** Time-frequency plots during invalidly minus validly cued single-grating trials. The ERSP is thresholded at *P* < 0.05 corrected for the number of electrodes (*n* = 9) using a nonparametric bootstrapping approach with 1,000 randomizations. **(D)** wPLI analysis for the frequency band from 15 to 20 Hz, indicating the effect of invalidity on functional connection between IPS and SPL. A positive *y* value means that the phase lead is in the direction from A3 to A12, as mentioned in the title of the plot, a negative *y* value that it goes in the opposite direction. The significance threshold was *P* < 0.05 corrected for the number of connections tested (*n* = 36) using a nonparametric bootstrapping approach with 2,000 randomizations.

Early ERP effects of invalidity occurred in SPL (Figure 3A: A3, B2; Figure 3B: A1, A3) and in the upper bank of posterior IPS (Figure 3A: A12; Figure 3B: A11–12, A16). In medial SPL (B2) the invalidity effect occurred as early as 163 ms following grating onset (Figure 3A). An ERP effect of invalidity was present in lateral SPL from 257 to 277 ms following target onset (Figure 3A: site A3). ITC within the theta band was increased following invalidly vs. validly cued targets as early as 200 ms following grating onset (A1 from 204 to 253 ms and A3 from 268 to 298 ms, respectively) (Figure 3B). An interaction analysis between the side of the relevant grating and the invalidity effect did not reveal any significant ERP interaction effects.

There were also later effects which are less likely to be related to the spatial shift *per se* (Müller et al., 1998; Figures 3B,C). Starting around 436 ms after grating onset, an invalidly cued trial elicited greater synchronization in the high gamma range than a valid trial (Figure 3C: electrode sites A17–18) and more desynchronization in the high beta range (Figure 3C: electrode sites A11, A16–17) in IPS. This effect did not depend on the target side.

Invalidity was also associated with a significant change in connectivity between IPS and SPL: From around 400–568 ms there was a transient phase lead of SPL with respect to IPS following an invalidly cued target compared to a validly cued target, suggesting that for a brief period of time, activity in SPL was preceding IPS activity (Figure 3D: A3 with respect to A12).

### Selection between competing stimuli

**Figure 4:**
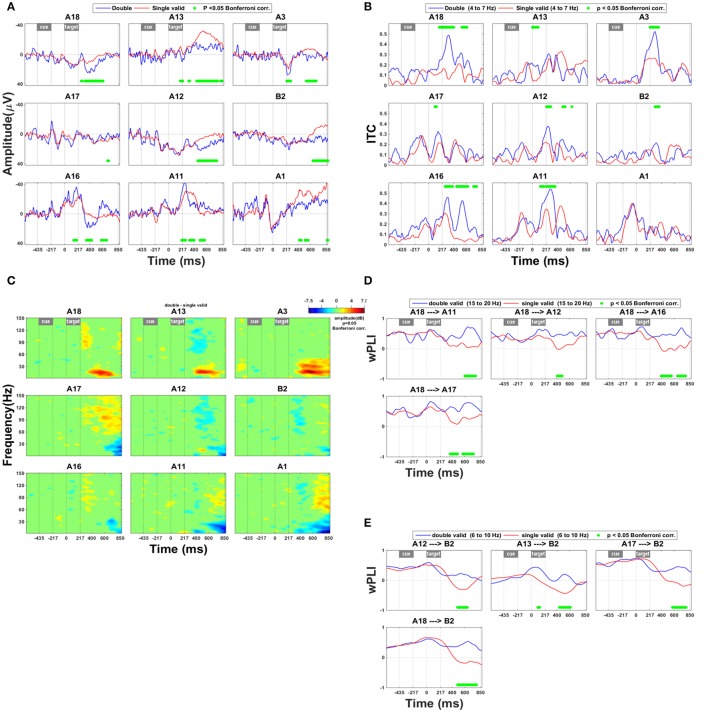
**Effect of the presence of a competing stimulus. (A)** ERP during competition trials compared to validly cued single-grating trials. Significant deficits following target onset between validly cued and invalidly cued trials are marked by a green bar. The significance threshold is set at *P* < 0.05 corrected for the number of electrodes during a minimum continuous period of 10 ms. The plots for the different electrodes are placed in accordance with their position on the cortical surface (Figure 1A). **(B)** Inter-trial coherence during competition trials compared to validly cued single-grating trials. The significance threshold is set at *P* < 0.05 corrected for the number of electrodes (*n* = 9) using a nonparametric bootstrapping approach with 1,000 randomizations. **(C)** Time-frequency plots during competition trials minus validly cued single-grating trials. The ERSP was thresholded at *P* < 0.05 corrected for the number of electrodes (*n* = 9) using a nonparametric bootstrapping approach with 1,000 randomizations. **(D)** wPLI analysis indicating the effect of competition trials compared to valid single-grating trials on functional connection between anterior and posterior IPS in the frequency band 15–20 Hz. The significance threshold was *P* < 0.05 corrected for the number of connections tested (*n* = 36) using a nonparametric bootstrapping approach with 2,000 randomizations. **(E)** wPLI analysis indicating the effect of competition trials compared to valid single-grating trials on the functional connection between IPS and SPL in the frequency band 6–10 Hz. Same significance threshold as in **(D)**.

The effects of a competing distracter differed drastically from the invalidity effects in their time course, spatial distribution, and spectral power (Figure 4). Along the lower and upper bank of IPS (A12–13, A18) and in SPL (A3, B2) the presence of a competing distracter caused a prolonged effect on the ERP from around 310 ms onwards (Figure 4A). The presence of a competing distracter was associated with synchronization in the high gamma range (Figure 4C: A11, A17, and A1). Rather uniquely for the competition trials, in the anterior electrodes in IPS (A18, A13) and SPL (A3) there was less desynchronization in the high beta band compared to single grating trials (Figure 4C). The distribution of this beta band effect co-localized with that of the ERP effect shown in Figure 4A. When a competing distracter was present, the directed influence of anterior on middle IPS remained positive for a longer period of time. This suggests that the effect of anterior IPS to middle IPS regions was more prolonged in competition trials compared to single-grating valid trials (Figure 4D). Within the 6–10 Hz frequency band, the directed influence of IPS on SPL also remained positive for a longer period of time (Figure 4E).

## Discussion

The current study provides for the first time the electrophysiological signature of the spatial shifting signal in response to an invalidly cued spatial cueing trial in SPL. The invalidly cued target requires a spatial shift, triggered by the stimulus appearing at an unexpected location. We propose that this spatial shift underlies the early SPL effects. The competition trials require selection between two competing stimuli based on short-term memory of the direction of the prior spatial cue. In the past, we had proposed that the activation of IPS during the competition trials was related to the attentional priority map as described in LIP (for review see Vandenberghe and Gillebert, 2009). The long latency of the effect appears to exclude that IPS plays a role in setting the attentional weights in an early selection stage (Bundesen et al., 2005). The late IPS response may reflect attentional priority setting in a late selection stage or response decision processes.

### ECoG effects of invalidity in SPL

Around 200 ms, and as early as 167 ms, an ERP effect was found in SPL in response to an invalidly cued target compared to a validly cued target. Around the same time and at approximately identical electrodes, an ITC effect was present that was congruent with the ERP effect. The congruency between the ERP and the ITC effect strengthens the evidence that the invalidity effect in SPL is robust. Overall this timing is of the same order as that described for the behavioral effect of exogenous reorienting (Müller and Rabbitt, 1989). wPLI measures an entirely different dimension of the data, namely the phase lag between electrodes. Approximately 200 ms following the ITC/ERP effect in SPL there is a reversal of the direction of the phase lag so that SPL leads IPS. Because of this long latency the reversal of the phase lag cannot be directly related to the mechanism of the spatial shift, which occurs earlier, both behaviorally and electrophysiologically. The reversal of the phase lag may be related to a cognitive process after the shift, e.g., decision-related processes, a re-setting of the attentional set, or the increased cognitive control required by an unpredicted event.

The Posner spatial cueing paradigm has been studied using ECoG in one previous study which principally focussed on changes in coherence of the signal within and across network nodes (Gunduz et al., 2012; Daitch et al., 2013): Following an invalidly cued target, theta synchronization was seen in both the dorsal and the ventral attention network. This has been termed the theta band reorienting response (Daitch et al., 2013). ITC analysis of the current dataset allowed us to localize a theta reorienting response with higher anatomical specificity to the SPL in invalid compared to valid trials as well as the upper bank of the posterior segment of IPS. The early theta reorienting response in SPL was specific for invalid trials (A1: Figure 3B vs. Figure 4B) and was not present during competition trials at that recording site. In ERP, reorienting to distracters that share task-relevant features with the target is associated with changes in the theta frequency band (Chang et al., 2016). In stroke patients with left spatial neglect, the attentional benefit induced by task-relevant features of distracters upon the processing of targets is diminished (Ptak and Schnider, 2010). This reduction is associated with a reduction of theta band connectivity within the structurally preserved dorsal attention network (Fellrath et al., 2016). Both task- and stimulus-driven factors may also play a role in the current experiment since the spatial shift to an invalidly cued target is triggered by the grating appearing at an unexpected location and the shift also matches the task goal. The theta reorienting effect therefore most likely reflects both stimulus-driven and task-driven attentional reorienting integrated. Although the spatial shift following the cue is driven by a central arrow, the reorienting during the target phase is partly driven exogenously by the appearance of the grating at the uncued location.

Based on prior evidence (Gunduz et al., 2012; Daitch et al., 2013) and in order to limit the number of comparisons we restricted the ITC analysis to the theta band. Electrophysiological studies based on surface EEG or magnetoencephalography have demonstrated alpha band desynchronization contralateral to the focus of attention in bilateral posterior sensors at 300–600 ms following cue onset as well as increases in alpha power contralateral to the ignored stimuli (Rihs et al., 2009). This alpha band desynchronization is considered a marker of allocation of spatial attention (Wyart and Tallon-Baudry, 2008; Capotosto et al., 2009; Hong et al., 2015). Alpha desynchronization occurs principally at occipital sensors outside the cortical surface covered in the current study and also relatively late with respect to the timing of the delay phase of the current study.

According to one of the most influential contemporary models of spatial attention in the human brain, the spatial reorienting deficit during invalidly cued trials in the Posner spatial cueing paradigm relates principally to inferior parietal lesions of the ventral attention network, most notably the right angular gyrus and TPJ (Corbetta and Shulman, 2002). The current study provides information about the contribution of superior parietal cortex to spatial attention. It demonstrates an early effect of spatial shifting in SPL. An important outstanding question is how the processes mediated by SPL in invalidly cued trials relate to those performed by the inferior parietal areas during spatial shifts, such as cytoarchitectonic area PF (Gillebert et al., 2013), and how the timing differs between these regions. Further ECoG studies with wider coverage would be needed to address this question. Ischemic lesions of SPL that spare IPS structurally and functionally are extremely rare. A case with bilateral damage to the SPL (MC) had a severe deficit in invalidly cued trials while performance on competition trials was relatively preserved (Vandenberghe et al., 2012). The nonhuman primate homolog of the SPL region activated during spatial shifts has recently been identified as area V6/V6a (Caspari et al., 2015). The structural and functional connections between the SPL regions involved in shifting and the inferior parietal or prefrontal cortex are a topic of ongoing research. Insight into these connections and the differences with IPS will be required in order to integrate the shifting-related activity in SPL into network models of spatial attention (Bartolomeo et al., 2012). It is also important to note that the recordings were limited to the left hemisphere and that the link between the current findings and the clinical phenomenon of right-hemispheric neglect (as opposed to visual extinction) is probably weak.

The absence of a directional effect in SPL is in full agreement with all previous studies in humans and in nonhuman primates that the shifting effect in SPL is not specific for the direction of the spatial shift (Vandenberghe et al., 2001; Yantis et al., 2002; Molenberghs et al., 2007; Caspari et al., 2015). In these studies, the shifting effect in SPL is systematically present in both hemispheres. We do not claim that the response in SPL explains the contralesional shifting deficit seen in the current patient or in patients with lateralized spatial-attentional deficits following stroke. In fact, the effect of direction of attention following lesions is almost certainly not mediated by SPL, but may, for instance, originate from topographically organized regions in IPS (Silver et al., 2005; Gillebert et al., 2011).

### Relation to visual neglect and the clinical symptom of extinction

The patient had no MRI-visible cortical lesions. The patient showed a contralesional shifting deficit and a contralesional deficit for the competition trials. A contralesional shifting deficit and a contralesional deficit on the competition trial does not imply neglect. In a previous study (Gillebert et al., 2011), among the 7 parietal lesion patients who had a contralesional shifting or a contralesional selection deficit in the hybrid spatial cueing paradigm, only two had pathological scores on the clinical tests of target cancelation or clinical extinction. In another study of the competition trials in 20 (sub)acute stroke patients, all four patients who had neglect scored pathologically on the competition trials but one subject had a contralesional deficit on the competition trial but normal scores on the clinical neglect tests (Molenberghs et al., 2008). Hence the computerized tests are more sensitive than the conventional clinical neglect tests. The right-hemispheric preponderance has been shown for the neglect syndrome but, as of yet, not for the current computerized tests. It is worth noting that in the canonical paper of the invalidity effect in parietal lesion patients by Posner et al. (1984), a contralesional shifting deficit was present in both left- and right-hemispheric lesion patients. Neglect is a more severe syndrome that consists of multiple components (for review see Vandenberghe et al., 2012). The spatial-attentional deficits measured by our tests are also present in neglect but are not sufficient to diagnose neglect. Patients who score normally on the clinical extinction test and who do not have neglect, may still have a contralesional shifting deficit and a contralesional competition effect on these computerized tests which are more sensitive for spatial-attentional deficits than the clinical tests for neglect (see Gillebert et al., 2011). Currently there is no evidence for hemispheric lateralization of the specific and subtle deficits detected by the hybrid spatial cueing paradigm, in contrast with neglect or visual extinction where there is a right-hemispheric dominance.

### Effects of cue direction during the delay phase

In the delay phase a direction-sensitive negativity occurred at the end of the delay in the more posterior IPS electrodes. The spatial distribution of the effect of direction of attention differed from that of the reorienting effect. This fits with nonhuman primate functional MRI (fMRI) data showing a clear dissociation between the effect of direction of attention (mainly localized to the Lateral Intraparietal area, among other regions) and the effect of shifting attention (mainly localized to V6/V6A) (Caspari et al., 2015). The timing of the cue direction effect may seem relatively late but is in agreement with the timing characteristics of the Early Directing Attention Negativity (EDAN) potential, a surface-EEG ERP deflection contralateral to the direction of attention (Harter et al., 1989; Yamaguchi et al., 1994; Nobre et al., 2000; Grent-'t-Jong and Woldorff, 2007; Simpson et al., 2011).

### ECoG effects in IPS of a competing distracter

The current study provides a unique insight into the time course of the IPS response to competing distracters. In the past, we proposed that the fMRI response in middle IPS to the presence of competing distracters reflects the compilation of the attentional priority map needed to prioritize between stimuli (Vandenberghe et al., 2005; Molenberghs et al., 2008). The ECoG data reveal that the latency of the IPS effect was more than 250 ms after the grating onset. Overall, this would rather suggest that the effect of stimulus competition in IPS mainly originates at a late-selection, postperceptual stage. It could be related to the low frequency (1 out of 6 trials) of the competition and invalid cueing trials compared to single valid trials, to the higher working memory demands of competition trials, e.g., related to a higher load (Gillebert et al., 2012) or to the higher endogenous selection demands of competition trials (Duncan, 2010). The data could still be compatible with a role in assigning attentional priorities to perceptual units at a late rather than an early selection stage. In an ECoG study of spatial attention using a different paradigm (Malhotra et al., 2009), ERSP in a time window from 400 to 600 ms revealed synchronization in the high gamma band during the more demanding attentional task (Park et al., 2016). Desynchronization occurred in the theta, alpha and beta band in superior parietal cortex during the spatial attention task from 400 to 800 ms bilaterally (Park et al., 2016). In the current study, ERSP revealed in IPS an increase in high gamma synchronization in response to invalidly cued targets as well as as to competition trials, similar to the effect described by Park et al. (2016).

### Study limitations

The study limitations are mainly related to the ethical restrictions imposed by the clinical utility that is required for all aspects of the procedure. Foremost, this is a single-case report. In our opinion, the unique nature of the ECoG data with its supreme spatial and temporal resolution compensates for the single-case nature. Second, interictal epileptic activity may have interfered with the measurements. The clinical indication for the ECoG measurements implies that the cortical tissue from which recordings are made may be dysfunctional and one should bear this in mind when drawing inferences regarding healthy intact neocortical tissue. Third, human IPS is a very convoluted sulcus with a large part buried deeply within the sulcus itself. EEG recordings mainly detect signal from the cortical surface and are less sensitive for activity arising from within the depth of the sulcus. This is also the case for ECoG which uses a subdural grid instead of depth electrodes.

## Conclusion

To conclude, the current study reveals the electrophysiological signature of spatial-attentional shifting in SPL. In line with previous nonhuman primate studies (Caspari et al., 2015) the effect of spatial shifting is anatomically dissociable from the effect of the direction of attention, and also from the effect caused by the presence of competing stimuli. In IPS the effect of spatial cue direction in more posterior electrodes and the long-latency response to the presence a competing distracter in more anterior electrodes reconciles a spatial interpretation of the role of IPS with its contribution to general-purpose attentional control processes (Duncan, 2010).

## Author contributions

MS, EG, PD, RV: substantial contributions to conception and design of the work and the acquisition, analysis and interpretation of the data, and drafting and rewriting the manuscript. LS, TT, WV: Substantial contribution of the acquisition and interpretation of the data to the intellecutal content of the paper. All authors provided final approval of the version to be published and agree to be accountable for all aspects of the work.

## Funding

RV is a Senior Clinical Investigator of the Research Foundation Flanders (FWO). MS is supported by Klinisch Onderzoeksfonds UZ Leuven and an FWO Clinical PhD fellowship (1701413N). This research was funded by KU Leuven Grant OT/12/097, FWO G0A0913N, and Federaal Wetenschapsbeleid Belspo Inter-University Attraction Pole Grant P7/11.

### Conflict of interest statement

The authors declare that the research was conducted in the absence of any commercial or financial relationships that could be construed as a potential conflict of interest.

## Abbreviations

CSD: cross-spectral density
ECoG: electrocorticography
EEG: electroencephalography
EOG: electrooculography
ERP: event-related potential
ERSP: event-related spectral perturbation
IPS: intraparietal sulcus
ITC: intertrial coherence
MNI: Montreal neurological institute
MRI: magnetic resonance imaging
PET: positron emission tomography
SPL: superior parietal lobule
TPJ: temporoparietal junction
wPLI: weighted phase lag index.
